# PReS-FINAL-2340: A pathogenic role for S100A8/A9 in experimental autoimmune arthritis

**DOI:** 10.1186/1546-0096-11-S2-P330

**Published:** 2013-12-05

**Authors:** C Kessel, J Roth, D Foell, T Vogl

**Affiliations:** 1 Pediatric Rheumatology & Immunology, Clinical-Translational Innate Immunity Research, University Children's Hospital, Münster, Germany; 2 Institute of Immunology, Münster, Germany; 3 Center for Interdisciplinary Clinical Research, University of Münster, Münster, Germany

## Introduction

**D**amage **A**ssociated **M**olecular **P**attern molecules (DAMPs) like HMGB1 have been demonstrated to be involved in pathological processes triggering and perpetuating autoimmune arthritis by attraction and activation of immune cells. The phagocyte-specific S100 proteins A8 and A9 (**M**yeloid **R**elated **P**rotein 8 and 14, respectively) are expressed by monoyctes and granulocytes and can likewise operate as DAMPs by signalling through TLR4 once released into the extracellular space.

## Objectives

S100A8/A9 has initially been identified in context with rheumatoid arthritis (RA). Generally, the proteins have a high relevance as inflammatory biomarker in various arthritic conditions, such as juvenile idiopathic arthritis (JIA). The proteins' involvement in triggering or perpetuating pathomechanisms in autoimmune polyarthritis is albeit discussed controversially. We set out to carefully re-investigate this task and dissect possible contributions of S100A8/A9 to arthritis pathogenicity.

## Methods

Collagen induced arthritis was induced in C57BL/6 and S100A9^-/- ^mice, the latter lacking both S100A9 and its binding partner S100A8 at protein level. Disease progression was monitored by both clinical score and serological anti type II collagen (CII) autoantibody levels. Splenic T cell responses upon CII re-stimulation were studied in presence or absence of S100 protein priming of either whole splenocytes or bone marrow derived macrophages (BMDMs).

## Results

S100A9^-/- ^mice were almost completely protected from collagen induced arthritis (CIA). As observed throughout all experiments, this is accompanied by a strikingly significant decrease in anti type II collagen antibody titers compared to C57BL/6 wild type animals. Compared to wt-T cells, splenic S100A9^-/- ^T cells respond poorly to CII re-stimulation. This phenotype can be partly reversed, if S100A9^-/- ^splenocytes or macrophages (BMDMs), as the dominant CII antigen presenting cells, are primed with either S100A8 or S100A8/A9.

## Conclusion

In an autoimmune arthritis mouse model S100A8/A9 can apparently operate at the interface between innate and adaptive immunity, possibly by modulating antigen presentation capacities of macrophages and thus affecting CD4^+ ^T cell stimulation and downstream autoantibody production. Beyond their role as inflammatory biomarkers in arthritis S100A8/A9 can therefore likely trigger and promote autoimmunity. An active contribution to a distorted cross-talk between innate and adaptive immune mechanisms, as potentially in place in poly- or oligoarticular JIA, can be suggested.

(*DF, TV: equal contribution*)

## Disclosure of interest

None declared.

